# Miocene Climate and Habitat Change Drove Diversification in *Bicyclus*, Africa’s Largest Radiation of Satyrine Butterflies

**DOI:** 10.1093/sysbio/syab066

**Published:** 2021-08-07

**Authors:** Kwaku Aduse-Poku, Erik van Bergen, Szabolcs Sáfián, Steve C Collins, Rampal S Etienne, Leonel Herrera-Alsina, Paul M Brakefield, Oskar Brattström, david J Lohman, Niklas Wahlberg

**Affiliations:** Department of Zoology, University of Cambridge, Cambridge, UK; Biology Department, University of Richmond, Richmond, VA, USA; Department of Life and Earth Sciences, Perimeter College, Georgia State University, GA, USA; Biology Department, City College of New York, City University of New York, NY, USA; Department of Zoology, University of Cambridge, Cambridge, UK; Department of Systematic and Evolutionary Botany, University of Zurich, Zurich, Switzerland; Institute of Silviculture and Forest Protection, University of Sopron, Sopron, Hungary; African Butterfly Research Institute, Westlands, Nairobi, Kenya; Groningen Institute for Evolutionary Life Sciences, Groningen, The Netherlands; School of Biological Sciences, University of Aberdeen, Aberdeen, Scotland, UK; Department of Zoology, University of Cambridge, Cambridge, UK; Department of Zoology, University of Cambridge, Cambridge, UK; African Butterfly Research Institute, Westlands, Nairobi, Kenya; University of Glasgow, School of Life Sciences, Glasgow, Scotland, UK; University of Glasgow, Institute of Biodiversity, Animal Health and Comparative Medicine, Glasgow, Scotland, UK; Biology Department, City College of New York, City University of New York, NY, USA; Ph.D. Program in Biology, Graduate Center, City University of New York, NY, USA; Entomology Section, National Museum of Natural History, Manila, Philippines; Department of Biology, Lund University, Lund, Sweden

## Abstract

Compared to other regions, the drivers of diversification in Africa are poorly understood. We studied a radiation of insects with over 100 species occurring in a wide range of habitats across the Afrotropics to investigate the fundamental evolutionary processes and geological events that generate and maintain patterns of species richness on the continent. By investigating the evolutionary history of *Bicyclus* butterflies within a phylogenetic framework, we inferred the group’s origin at the Oligo-Miocene boundary from ancestors in the Congolian rainforests of central Africa. Abrupt climatic fluctuations during the Miocene (*ca.* 19–17 Ma) likely fragmented ancestral populations, resulting in at least eight early-divergent lineages. Only one of these lineages appears to have diversified during the drastic climate and biome changes of the early Miocene, radiating into the largest group of extant species. The other seven lineages diversified in forest ecosystems during the late Miocene and Pleistocene when climatic conditions were more favorable—warmer and wetter. Our results suggest changing Neogene climate, uplift of eastern African orogens, and biotic interactions have had different effects on the various subclades of *Bicyclus*, producing one of the most spectacular butterfly radiations in Africa. **[**Afrotropics; biodiversity; biome; biotic interactions; Court Jester; extinction; grasslands; paleoclimates; Red Queen; refugia forests; dependent-diversification; speciation.]

Understanding the processes that promote diversification and their role in shaping current species distributions is a central theme in evolutionary ecology (Ricklefs 2006; Schluter 2009; Nosil 2012). The many proposed drivers generating biodiversity can generally be grouped into two classes. Red Queen models focus on biotic or intrinsic factors in which ecological interactions among species or responses to the environment influence diversification (Van Valen 1973). These may include competition, predation, changes in physiological tolerances, and adaptability to change or unfavorable conditions. In Court Jester models, abiotic or extrinsic factors drive diversification. These factors may involve large-scale perturbations in the physical environment such as climate change or major shifts in geology, such as mountain building or rift formation (Barnosky 2001; Benton 2009). These two classes of models are, however, not mutually exclusive, and could operate in unison or in succession (Erwin 2009; Ezard et al. 2011; Ezard et al. 2016).

Recent advances in mechanistic modeling of macroevolution allow the relative effects of intrinsic biotic interactions and extrinsic abiotic factors on diversification to be evaluated by modeling diversification rates as a function of time, diversity, environmental changes, and character states using time-calibrated phylogenies (Stadler 2011; Etienne et al. 2012; Condamine et al. 2013, 2018a; Morlon et al. 2016, 2020; Lewitus and Morlon 2018; Herrera-Alsina et al. 2019) but see Louca and Pennell (2020). Using these tools, evolutionary histories and inferred diversification mechanisms of many taxa have been identified (e.g., Claramunt and Cracraft 2015; McGuire et al. 2014; Toussaint et al. 2014; Lagomarsino et al. 2016; Condamine 2018). However, there are few studies aimed at unravelling the mechanisms and historical events that generated and maintain biodiversity in the Afrotropics, especially for clades that are endemic to the region. Most evolutionary investigations of Africa’s biodiversity have focused on plants (Fjeldså and Lovett 1997; Schnitzler et al. 2011; Dagallier et al. 2020), vertebrates (Kappelman et al. 2003; Portik et al. 2017; Pozzi 2016), and aquatic organisms (Seehausen 2006; Wilson et al. 2008). In contrast, large invertebrate groups, which constitute the bulk of the continent’s fauna, have been largely neglected (but see Kergoat et al. 2018).

*Bicyclus* Kirby 1871, an African-endemic butterfly genus, is an ideal taxon for evaluating the contributions of biotic and abiotic factors in the generation and maintenance of Afrotropical biodiversity. With 102 currently recognized species (Aduse-Poku et al. 2017), *Bicyclus* is by far the most diverse and species-rich satyrine genus in Africa. The genus is found throughout the sub-Saharan parts of the continent, occupying a wide range of habitats on the mainland. A single species, *Bicyclus anynana,* extends its range to the Comoros and Socotra islands in the Indian Ocean. Although the majority of *Bicyclus* species are restricted to forests, there are several savannah or savannah-woodland specialists, as well as generalist species occupying both forest fringes and opened habitats (Condamin 1973; Larsen 2005). In addition, several species are restricted to lowland, submontane, or montane habitats. It remains unclear when this habitat specialization evolved and what processes facilitated the ecological differentiation. Most *Bicyclus* larvae are believed to feed on grasses (Poaceae) (Larsen 2005), and it is likely that they underwent rapid diversification during the Miocene when African grasslands expanded (Jacobs 2004; Uno et al. 2016). Diversification might have been facilitated by changes in host plant breadth (Nokelainen et al. 2016; van Bergen et al. 2016) in combination with an appearance of or dispersal to new environments with differing diversity-dependent diversification processes (Etienne and Haegeman 2012).

Alternatively, large-scale biodiversity patterns can also be influenced by the abiotic environment (Benton 2009). Africa has experienced significant changes in climate and geology during the Cenozoic. For example, temperatures rose during the mid-Miocene Climate Optimum (MMCO) 15–17 Ma (Bohme 2003) and became cooler throughout the mid-Miocene starting *ca*. 14 Ma (Zachos et al. 2008). Moreover, the orogeny of the East African Plateau also occurred during the Miocene (Roberts et al. 2012; Wichura et al. 2015), and is believed to have drastically altered atmospheric circulation in the region (Sepulchre et al. 2006), intensifying African aridification and vegetation change (Linder 2017). These past fluctuations in climate, geomorphology, and biome composition are expected to have also influenced the diversity and geographic distribution of plant-feeding insects.

Like many tropical fruit-feeding butterflies, *Bicyclus* can be collected readily with bait traps, and the genus has therefore been the focus of many ecological and conservation studies (Molleman et al. 2006; Bossart and Opuni-Frimpong 2009; Aduse-Poku et al. 2012). As a consequence, there is a plethora of ecological data on behavior, habitat preferences, host plant use, phenology, and geographical distribution available for many species. Further, *B. anynana* has been a model species for the study of evolution, behavior, and development (Brakefield 2001; Beldade and Brakefield 2002; Brakefield et al. 2009; Rivera-Colon et al. 2020; Matsuoka and Monteiro 2021) for almost four decades. Species boundaries and phylogenetic relationships within the genus were recently clarified with genetic data (Aduse-Poku et al. 2017), so the stage is set for comparative studies aimed at understanding the mechanisms underlying the evolutionary success of the genus. Here, using genetic data, species-specific ecological and geographic distribution information, and a series of analytical macroevolutionary models, we explore the evolutionary history of *Bicyclus* and examine the roles of host plant interactions, climate, and mountain building on the radiation of this diverse, African butterfly group.

## Materials and Methods

### Taxon Sampling and Genomic Data Set

Of the 102 currently recognized Bicyclus species, 94 (92%) were included in this study (Supplementary Appendix S1 available on Dryad at http://dx.doi.org/10.5061/dryad.qz612jmcb). The included samples covered all 16 currently recognized species-groups (Aduse-Poku et al. 2017). We also included the two recognized species of its sister genus, *Hallelesis* (*H. halyma* and *H. asochis*) and 11 other closely related satyrinae taxa as outgroups, selected on the basis of evolutionary relationships recovered in two earlier higher-level phylogenetic studies (Espeland et al. 2018; Chazot et al. 2019). We used a total of ten protein-coding loci: one mitochondrial (cytochrome c oxidase subunit I, COI) and nine nuclear (carbamoyl phosphate synthetase domain protein, CAD; ribosomal protein S5, RpS5; ribosomal protein S2, RpS2; wingless, wgl; cytosolic malate dehydrogenase, MDH; glyceraldehyde-3-phosphate dehydrogenase, GAPDH; elongation factor 1 alpha, EF-1$\alpha $; and arginine kinase, ArgKin and isocitrate dehydrogenase, IDH) (Wahlberg and Wheat 2008).

Most sequences used in this study were obtained from Aduse-Poku et al. (2017); additional sequences from six taxa were obtained using the protocols described in that study. All sequences were aligned manually using BioEdit 7.2 (Hall 1999) with properties and reading frames of protein-coding genes examined in MEGA X 10.0.5 (Kumar et al. 2018). Individual gene trees were first generated using IQ-TREE 1.6.11 (Nguyen et al. 2015) to check for contamination and sequence quality. Cleaned sequences were then concatenated to produce a final matrix of up to 7735 aligned nucleotides from 107 taxa (Supplementary Appendix S1 available on Dryad).

### Phylogenetic Reconstruction

Phylogenetic relationships were inferred under a maximum-likelihood (ML) framework with IQ-TREE 2.0.6 (Minh et al. 2020). The concatenated 10 protein-coding loci data set was first partitioned by locus and codon position, resulting in 30 initial partitions. The best-fit partitioning scheme and models of nucleotide substitution were estimated simultaneously using the greedy algorithm in ModelFinder (Kalyaanamoorthy et al. 2017), with the optimal models chosen based on corrected Bayesian Information Criterion. The best-fit models of nucleotide substitution were determined across all available models in IQ-tree, including the FreeRate model ($+$R; Soubrier et al. 2012), which relaxes the assumption of gamma distributed rates using the function MFP$+$MERGE.

We assessed nodal support of recovered relationships using 1000 ultrafast bootstrap replicates, UFBoot (Hoang et al. 2018), and the Shimodaira–Hasegawa approximate likelihood ratio test, SH-aLRT (Guindon et al. 2010). To overcome model violations inherent to UFBoot calculations, the “-bnni” command was added to improve the search for each UFBoot replicate tree. We also implemented the transfer bootstrap expectation (TBE) method (Lemoine et al. 2018) in IQ-TREE by invoking the function –tbe after 500 standard Felsenstein bootstraps. TBE provides a better measure of deep branch repeatability, or robustness, even when the phylogenetic signal is moderate (Lemoine et al. 2018). Nodes with SH-aLRT $\ge $ 80, UFBoot $\ge $ 95 and TBE $\ge $80 are considered strongly supported and moderately supported when SH-aLRT $\ge $ 80 or UFBoot $\ge $ 95 or TBE $\ge $80.

### Divergence Time Estimation

We estimated divergence times in a Bayesian framework using BEAST 1.10.4 (Suchard et al. 2018). Similar to the phylogenetic analysis, the concatenated sequence data were first partitioned by locus and codon position. The best-fit partitioning scheme and models of substitution were determined in PartitionFinder 2.1.1 (Lanfear et al. 2017) using the greedy algorithm, with linked branch lengths in the computation of likelihood scores. The best-fit model was selected based on the lowest AIC across all models included in BEAST (option models $=$ beast). A relaxed molecular clock was used for the molecular dating, allowing branch lengths to vary according to an approximate continuous time Markov chain rate reference prior (Ferreira and Suchard 2008). To examine the effect of our chosen priors on inferred divergence time estimates, we compared the performance of four analyses employing different models by estimating marginal likelihood estimates (MLE) using path-sampling and stepping-stone sampling with 1000 path steps. Each chain ran for one million generations with log likelihood sampling every 1000 cycles (Xie et al. 2011; Baele et al. 2013). The models included either one or two clocks: one for the mitochondrial partitions and one for the nuclear partitions. For each of these clock models, we also tested different tree models using a Yule or a birth–death model (resulting in four clock tree models), with the substitution model parameters for each partition estimated separately.

There are no known fossils of *Bicyclus* or closely related taxa, so we relied on secondary calibration points from the large-scale fossil-based dating framework of (Espeland et al., 2018) inferred for the butterfly superfamily Papilionoidea, where the crown of the subfamily Satyrinae was estimated to be 54.1 Ma (95% CI 40.7–67.4 Ma). Chazot et al. (2019) independently (but using several of the same fossils) estimated the crown age of Satyrinae to be 54.4 Ma (95% CI 46.0–64.8 Ma). However, the topologies within Satyrinae differ between the two studies. Given the relatively higher nodal supports of Espeland et al. (2018), we selected two additional calibration points from that study with two uniform priors encompassing the 95% credibility intervals for our tree calibration analyses. These nodes correspond to the split between the tribes Morphini and Brassolini and their most recent common ancestor (MRCA): subtribes Lethina and Mycalesina (Supplementary Appendix S2 available on Dryad).

BEAST dating analyses were run for 50 million Markov chain Monte Carlo (MCMC) generations with model parameters and tree sampled every 1000 generations, yielding a total of 50 000 samples per analysis. Convergence and performance of the MCMC were assessed using Tracer 1.7.1 based on the ESS threshold values of >200 for each parameter (Rambaut et al. 2018). Following a 25% burn-in, the program TreeAnnotator 1.10.4 (included in the BEAST package) was used to summarize the information (i.e., nodal posterior probabilities, posterior estimates and highest posterior density, HPD limits) from the post burn-in trees into a single maximum clade credibility (MCC) tree.

### Biogeographic Analyses

Ancestral ranges were estimated using the dispersal-extinction-cladogenesis (DEC) model (Ree and Smith 2008) implemented in the R package BioGeoBEARS 1.1.2 (Matzke 2018) with the Bayesian time-calibrated MCC tree with outgroups removed. The DEC model infers dispersal-mediated range expansion and extinction-mediated range contraction. The probability of either event occurring along a particular branch is proportional to the length of that branch and the instantaneous transition rates between geographic areas. The DEC model requires an ultrametric dated phylogeny and the geographic distributions of extant species. Due to their sometimes closely similar wing patterns and variable seasonal forms, *Bicyclus* are frequently misidentified in books, databases, and museum collections. Therefore, we only used data for which voucher specimens or photos were available to verify the validity of individual records. When using literature records, we only relied on sources for which we personally knew the identification skills of the author(s). We screened the collections of nine museums (ABRI Nairobi, CEP-MZUJ Krakow, EMUL Lund, MNHB Berlin, MRAC Tervuren, NHMUK London, NHMW Vienna, NHRS Stockholm, OUMNH Oxford, SMNS Stuttgart, and ZFMK Bonn), and the personal collections of Oskar Brattström, Thomas Desloges, Szabolcs Sáfián, Robert Tropek, Robin van Velzen, and Robert Warren. Online sources that provided clear photos of specimens were also used—primarily inaturalist.org and several Facebook groups. By supplementing these location data with information from two reliable published sources (Condamin 1973; Larsen 2005), we extrapolated the present geographic ranges of each species as occurring in one or more of these seven biogeographic regions following the borders defined by Linder et al. (2012): A, Saharan Region; B, Guinea Region; C, Congolian Region; D, Shaba Region; E, Zambezian Region; F, Southern African Region; and G, Ethiopian-Somalian Region (Fig. 2).

We ran BioGeoBEARS analyses with three dispersal rate scalar matrices. The first analysis (M0) assumed that dispersal between any two geographic regions was equally probable and used a single dispersal rate scalar value throughout time. The other two dispersal rate scalar matrices presumed that dispersal events between adjacent areas were not scaled or penalized (dispersal d $=$ 1.0), but dispersal events between nonadjacent areas were. The first constrained model (M1), applied a scalar of 0.75 (i.e., a penalty of 0.25) to dispersal events between areas separated by either a third region or by an oceanic barrier. A scalar of 0.50 was applied to dispersal events between areas separated by two regions. Penalties were summed as pairwise areas became progressively more distant from each other such that the farthest two regions (e.g., Guinean and Southern African) had a scalar of 0.25 (Supplementary Appendix S3a available on Dryad). In the second constrained model (M2), we estimated the scalar multiplier as $m^{n}$ (where $m$ < 1 and $n =$ number of regions separating the regions compared) by optimizing the BioGeoBEARS output for the loglikelihood (Supplementary Appendix S3b available on Dryad). The maximum number of areas per ancestral state was set to seven. Finally, to quantify the relative role of dispersal and vicariance on diversification, we implemented 1000 stochastic biogeographic mappings using the best-fit DEC model. From these mappings we obtained the overall probabilities of the anagenetic and cladogenetic events conditioned on the geographic distributions, the phylogeny, and the best-fit DEC model.

### Diversification Analyses

We first assessed whether the structure of the data (i.e., the topology of branching events) recapitulated the major taxonomic groups by applying a nonparametric method to our time-calibrated phylogeny (Lewitus and Morlon 2016). We computed the spectral density profile of the modified graph Laplacian of the phylogeny using the “SpectR” function of the R package RPANDA (Morlon et al. 2016) to determine the number of clusters in the tree. Using that number, we fit the eigengap heuristic 100 times to the consensus tree and to 200 trees randomly sampled from the posterior distribution of trees (after a 50% burnin-in) and estimated the percentage of times the clusters returned the same tips. We then cut the trees into the most frequently returned clusters or subclades. We then analyzed diversification patterns of the whole genus and each subclade separately.

To understand the relative contribution of intrinsic biotic interactions and extrinsic historical factors, we characterized diversification dynamics of each clade by fitting a series of macroevolutionary models to the data. To take into account uncertainty in tree topology and divergence time estimates, we fit all of the macroevolutionary models except the density-dependent diversification models first to the Bayesian consensus MCC tree, and then to 200 trees randomly sampled from the posterior distribution of the BEAST analysis (after a 50% burnin-in with outgroups removed). A sampling fraction of 0.92 was used for all analyses to take into account missing or unsampled taxa. We compared the fit of each model to the data using the AIC for each analysis. The AIC scores also allow computation of $\Delta $AIC (the difference in AIC between the model with the lowest AIC and the others) and Akaike weight ($w$AIC) which were subsequently used to select the best-fitting model (Burnham and Anderson 2002; Wagenmakers and Farrell 2004).

### Time-Dependent Diversification

We assessed variation in species diversification over time by performing a time-dependent diversification analyses using RPANDA (Morlon et al. 2016). We evaluated six models (the first two of which were null models) 1) speciation rate is constant over time with no extinction (Yule null model); 2) speciation and extinction rates are constant; 3) speciation rate varies over time with a single extinction rate that is constant over time; 4) speciation rate varies over time with no extinction; 5) speciation rate is constant but extinction rate varies over time; and 6) both speciation and extinction rates vary over time. Exponential dependence on time was assumed (Morlon et al. 2011). The models estimate current speciation and extinction rates and corresponding parameters measuring their variation over time up to the crown age. We also used the R package phytools 0.7-70 (Revell 2012) to generate lineage-through-time (LTT) plots to explore the temporal pattern of lineage accumulation within *Bicyclus* and its component clades.

### Environment-Dependent Diversification

We investigated the impact of environmental change on diversification using paleoenvironment-dependent models in RPANDA 1.9 (Condamine et al. 2013; Lewitus and Morlon 2018). We used proxies for four environmental variables that best characterize paleoclimatic change and are commonly associated with continental-scale macroevolutionary diversification 1) temperature (inferred from $\delta^{18}$O measurement, data taken from Zachos et al. 2008); 2) atmospheric CO$_{2\, }$(data from Beerling and Royer 2011); 3) $\delta ^{13}$C$_{organic}$ ($\delta^{13}$C value of the n-C$_{35}$ alkanes, data from Uno et al. 2016); and 4) plant fossil diversity (using Phanerozoic marine fossil diversity, data from Alroy 2010). Temperature is considered an important driver of biodiversity and evolutionary processes (Erwin 2009), and as such we examined the role of warming and cooling events on diversification. Changes in atmospheric CO$_{2}$ concentration over time likely affected photosynthesis and caused large-scale vegetation change that could impact insect evolution (Cerling et al. 1997; Tipple and Pagani 2007). Many *Bicyclus* feed on grasses, which are among the few plant taxa with C$_{4\, }$photosynthesis. Climate-induced grassland expansion may have increased the habitable area for grass-feeding insects, and the $\delta^{13}$C value of n-C$_{35}$ alkanes is considered the best proxy for inferring the presence of ancient C$_{4}$ grasslands in Cenozoic Africa because these values reflect global changes in organic carbon sequestration (Katz et al. 2005; Uno et al. 2016). Marine fossil diversity is considered a useful proxy for biological productivity, and the amount of land covered by plant biomass (Smol and Stoermer 2010) and was therefore used to test the impact of terrestrial habitats available for diversification.

By adapting the method of Condamine et al. (2018a), we developed four hierarchical models for each of the environmental proxies, and each of these models had exponential dependencies of speciation and extinction 1) speciation varies with the paleoenvironmental variable and no extinction; 2) speciation varies with the paleoenvironmental variable and constant extinction; 3) constant speciation, and extinction varies with the paleoenvironmental variable; and 4) both speciation and extinction vary with the paleoenvironmental variable. We then computed the AIC of each fit and plotted net diversification rate over time according to the model with the lowest AIC.

### Episodic Birth–Death (Tree-Wide) Diversification

To examine the impact of specific abiotic events on diversification, such as mass extinction, abrupt changes in plate tectonics, or climate change including the MMCO at 15–17 Ma, we used an episodic birth–death model implemented in the R-package TreePar 3.3 (Stadler 2011). This model assumes that rates of diversification (speciation and extinction) are constant over the entire tree but can vary within discrete time intervals (i.e., episodically). This allows detection of discrete changes in speciation and extinction rates concurrently affecting all lineages in a tree (i.e., tree-wide rate shifts) due to global mass extinction or environmental events affecting all lineages at once.

We used the function “bd.shifts.optim” in TreePar to compare the likelihood of five episodic birth–death models from zero (constant-rate model) to four diversification rate shifts during the evolutionary history of the group. For these analyses, we set the “grid” to 0.1 million years for a fine-scale estimation of rate shifts, the “end” as the crown age of the group and “start” as the present ($=$ 0 Mya). In addition to AIC scores and Akaike weights, we also calculated likelihood ratio tests (LRT) to select the best-fit between the different models allowing incrementally more shifts during the evolution of the group.

### Habitat-Dependent Diversification

We evaluated the effect of habitat changes as a potential driver of macroevolution using three trait-dependent diversification approaches. First, using both literature records (Condamin 1973; Kielland 1990; Libert 1996; Larsen 2005; Vande weghe 2009; Brattström et al. 2015; Brattström et al. 2016; Aduse-Poku et al. 2017; Coache et al. 2017) and the combined field experience of the authors, we categorized the habitat preferences of all taxa in our analyses into one of these categories: 1) forest-interior; 2) forest-fringe or intermediate-habitat; and 3) open-habitat or savannah. The taxa categorized as living in intermediate or forest-fringe habitats can be found in both forest and open habitats. Using the “ACE” function of the APE 5.5 package (Paradis et al. 2004) in R, we estimated the likelihoods of three transition models: equal rates (ER); symmetrical rates (SYM); or all rates different (ARD). A likelihood ratio test was used to determine the best-fitting model, which was used for all subsequent trait-dependent diversification analyses. Maximum likelihood estimation of ancestral habitat states was done using ACE to determine the marginal ancestral state reconstruction of the root and the conditional scaled likelihoods of all remaining nodes. However, to estimate the marginal probabilities for all nodes based on joint sampling, we used a different approach, namely stochastic mapping under a Bayesian framework. This was implemented in the R package phytools 0.7-70 (Revell 2012) using the “make.simmap” function. To account for branch length and topological uncertainty, we performed stochastic mapping using 100 replicates on 200 trees randomly selected from the posterior distribution of trees from the BEAST analyses, resulting in a total of 20 000 mapped trees. The number of transitions between character states and the proportion of time spent in each state were summarized using the “describe.simmap” function in phytools.

We then used a more comprehensive ML approach implemented in SecSSE 2.0.7 (Several Examined and Concealed States-Dependent Speciation and Extinction; Herrera-Alsina et al. 2019), to compare the likelihoods of models in which diversification rates depend on the type of habitat where species thrive (Examined Trait-Dependent [ETD]) with those in which diversification rates depends on a trait not examined in the analyses (Concealed Trait-Dependent [CTD]). In addition to ETD and CTD models, we also fit a Constant Rate (CR) model in which rates are homogenous over time and habitats. We extended the original SecSSE model by enabling two different modes of speciation: dual inheritance and single inheritance. In the former, the habitat preference of the ancestor is passed to both daughter species; similar to BiSSE (Maddison et al. 2007) and HiSSE (Beaulieu and O’Meara 2016). For instance, speciation of a forest-interior species will produce two forest-interior species. In single inheritance mode, only one daughter species inherits the same habitat preference as the ancestor, similar to ClaSSE (Goldberg and Igić 2012). In the context of our data, this would mean that a forest-interior species will produce, for example: 1) one forest-interior species and one open-habitat or savannah species or 2) one forest-interior species and one forest-fringe or intermediate-habitat species.

Because habitat preferences are not static over time, we defined five models that describe transitions across habitats. Under the *Unconstrained single rate* model, a given lineage can change its habitat preference to any of the other habitats, for example, a forest-interior species could become either an open-habitat or intermediate habitat species. All shifts in habitat preference are equally likely (i.e., they have the same rate). We also relaxed the assumption of equal rates in the *Unconstrained six rates* model in which all transitions could be different from one another. In the *Constrained single rate* model, we assumed that forest and open habitats are the end points of a gradient and species cannot change from one habitat to the other. Instead, lineages must transition through an intermediate state (e.g., a forest species cannot evolve directly into an open-habitat species but needs to first evolve into an intermediate forest-fringe habitat species). In this model, all events happen at the same rate. The *Constrained four rates* model is similar to the previous model because change in habitat preference requires transition through an intermediate habitat state, but the transition rates are all different (forest to intermediate habitat rate $\ne $ intermediate habitat to forest rate $\ne $ open-habitat to intermediate habitat rate $\ne $ intermediate habitat to forest rate). In the *Constrained specialist* model, we assumed that becoming a forest species or open-habitat species requires adaptations, and that such specialization evolves at a different rate evolving to be a generalist that is, an intermediate-habitat species. In the *Constrained openness* model, habitat preferences shift from densely vegetated habitats to open habitats and the reverse, with these two directions having different rates. In other words, the transition rates from forest (i.e., dense habitat) to intermediate and then open habitat species will all be the same but differ from transition rates in the opposite direction.

We set up 36 models that combine trait dependence (ETD, CTD, CR), speciation (Dual and Single Inheritance), and habitat evolution (*Unconstrained single rate*, *Unconstrained six rates*, *Constrained single rate*, *Constrained four rates*, *Constrained specialist,* and *Constrained openness*) and ran ML optimizations using three different starting points for each analysis to prevent finding only local optima. Because the number of free parameters varied across models, we compared their AICc scores. With the parameters that maximize the likelihood of the best supported model, we obtained the probability of habitat preference state at each internal node. This procedure is similar to ancestral state reconstruction, but a more conceptually accurate interpretation would be that the probability of habitat preference at a given node reflects the probability of that node producing descendant lineages with their observed (or inferred) habitat preferences given the model and its parameters. To further validate these SecSSE models, we used the speciation rates and transition matrix parameters of the best performing CTD and ETD models to simulate 30 data sets of phylogenetic trees for extant species with corresponding habitat preferences. Simulated time (crown age) was adjusted such that simulated trees were similar in size to the empirical one. We then fitted SecSSE CTD and ETD models to the simulated data sets in a manner similar to the main analysis. Afterwards, we compared the loglikelihood values of both models to find the number of simulations where the analysis chooses the right model. In other words, we counted the number of ETD-simulated data sets in which SecSSE found ETD as the best performing model. Similarly, we counted the number of cases where ETD was (erroneously) selected as the most likely model when in fact CTD was the generating model.

We also used diversity-dependent models (Etienne et al. 2012) to examine whether biotic interactions within *Bicyclus* influenced their diversification. Specifically, we investigated whether lineages diversified rapidly early in their evolutionary history and subsequently reached an equilibrium because ecological limits to their carrying capacities constrained diversification (Rabosky 2013). We explored the effect of diversity on speciation and extinction rates using the function “dd_ML” in the R-package DDD 4.4.1 (Etienne et al. 2012). We set up five diversity-dependent models in which: 1) speciation declines linearly with diversity without extinction (DDL); 2) speciation declines linearly with diversity and non-zero extinction (DDL$+$E); 3) speciation declines exponentially with diversity and non-zero extinction (DDX$+$E); 3) extinction increases linearly with diversity (DD$+$EL); and 5) extinction increases exponentially with diversity (DD$+$EX). We set the initial carrying capacity equal to the number of known species in the clade and used a set of three different starting points for each analysis.

We tested further whether Clade II (lineage 8) diversified under a different diversity-dependent scenario by decoupling its dynamics from Clade I in key innovation (KI) models (Etienne and Haegeman 2012) using the function “dd_KI_ML” in DDD. Specifically, we determined the timing of the decoupling (key innovation event) and whether the different diversity-dependent regimes differ only in their respective carrying capacities or also differed in their intrinsic speciation rate and extinction rates. Our KI models include models in which Clade II differs from Clade I 1) only in carrying capacity K (KI1); 2) in K and extinction rate $\mu $ (KI2); 3) in K and speciation rate $\lambda $, (KI3); and 4) in K, $\mu $, and $\lambda $ (KI4). Also, to ascertain whether the observed asymmetry between the two clades did not occur by chance and that the increased branching in Clade II is a result of a clade-wide shift in diversification rates affecting the entire tree, we implemented four additional shifting rate (SR) models using the function “dd_SR_ML” in DDD. The SR models we tested include a shift in K (SR1); in K and $\mu $ (SR2); in K and $\lambda $ (SR3); and in K, $\mu $, and $\lambda $ (SR4). The five, standard diversity-dependent models estimated above with the “dd_ML” function included the constant-rate (CR) models for comparison. To ensure sufficient convergence of the likelihood estimates, three independent analyses were performed for each model, each with different initial values for the diversification parameters. We fit the diversity-dependent DDD models to the Bayesian MCC tree. The results of the KI models and SR models cannot be compared directly because the KI models contain a lineage-specific shift, whereas the SR models contain clade-wide shifts. It has recently been shown that previous approaches for lineage-specific shifts were mathematically incorrect (Laudanno et al. 2020). The proposed correction employed here implies that SR and KI models cannot be directly compared, but we can, however, compare the KI models to a version of the CR and DDL models with such a single-lineage shift without actual change in parameters (called a dummy shift, KI0A, KI0B). We can also compare SR models to CR models, which enables us to indirectly compare KI and SR models.

Finally, to assess the relative contribution of time-specific global events including historic changes in climate, biotic interactions among conspecifics, and time-dependent processes on diversification, we compared the AIC scores of the best-fit models of each macroevolutionary analysis. We then computed the Akaike weights across the different macroevolutionary best-fit models to determine the relative roles of each process in the diversification of the group. The SecSSE models were excluded from this comparison because differences in the conditioning and normalization factors used in the likelihood computation made them not directly comparable with the other approaches (Stadler 2013). All other macroevolutionary analyses carried out in the study used the same data—branching times and conditioning for nonextinction of the descendants of the root lineage—and are therefore comparable (Stadler 2013; Condamine 2018a).

## Results

### Phylogeny and Divergence Time Estimates

**Figure 1.**
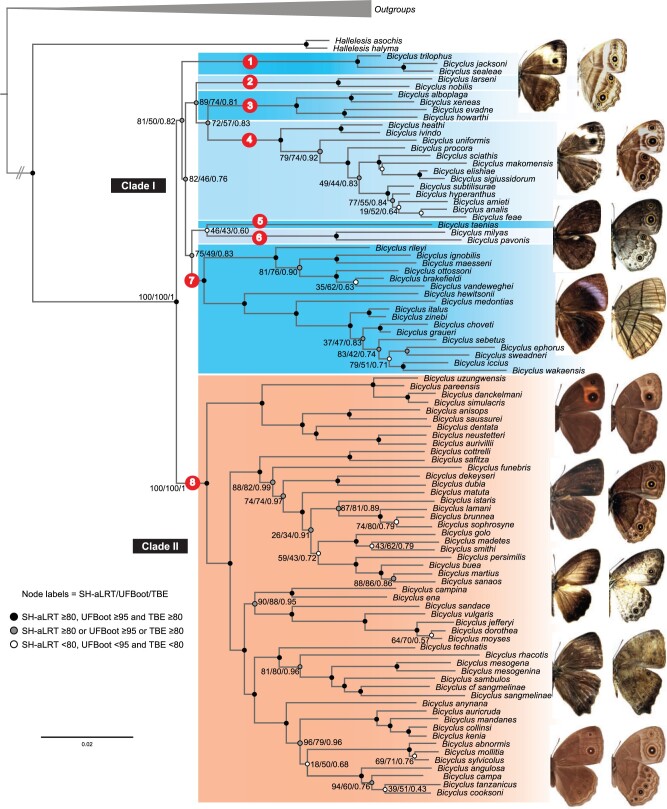
Best scoring ML phylogenetic tree of *Bicyclus* and its sister genus *Hallelesis* inferred with IQ-TREE based on a data set of 10 concatenated loci. The two principal clades are labeled Clade I and Clade II. The eight early-divergent lineages are labeled with numerals within red ovals. Node circles indicate nodal support values, where SH-aLRT, UFBoot, and TBE are Shimodaira–Hasegawa approximate likelihood ratio, ultrafast bootstrap tests, and transfer bootstrap expectation, respectively. *Hallelesis* and *Bicyclus* exemplars (from top to bottom): *H. halyma*, *B. xeneas*, *B. taenias*, *B. ephorus*, *B. uzungwensis*, *B. madetes*, *B. dorothea*, *B. sambulos*, and *B. anynana*. Pictures by Kwaku Aduse-Poku and Oskar Brattström.

The estimated tree topologies from the ML framework inferred with IQ-TREE and the Bayesian method dating analyses in BEAST were largely congruent (Fig. 1, Supplementary Appendix S4 available on Dryad). Dating analyses using different clocks and tree models estimated similar ages with broadly overlapping credibility intervals (Table 1). Based on the MLE comparison (Table 1), the analysis with two clocks and a birth-death model (T$_{2})$ was selected for all subsequent analyses. Our estimated phylogenetic hypotheses confirm that *Bicyclus* is monophyletic (SH-aLRT $=$ 100, UFBoot $=$ 100, TBE $=$ 1) and diverged from its sister genus *Hallelesis* around 24.9 Ma (95% HPD 20.5–29.1). The diversification of *Bicyclus* started about 5 Ma after the split of the MRCA of the group from *Hallelesis* in the mid-Miocene (20.2 Ma; 95% HPD 16.2–23.3). The initial events of cladogenesis within the crown group are inferred to have occurred rapidly and contemporaneously among eight major lineages between 19 and 17 Ma. The evolutionary relationships among these eight early-divergent lineages are largely unresolved (i.e., the nodes have low to moderate support) with relatively short branches (Fig. 1). Beyond the initial basal rapid divergence, the events of cladogenesis within the eight early-divergent lineages are recovered with high to moderate support. Seven of the eight early-divergent lineages form a clade, albeit with moderate nodal support (SH-aLRT $=$ 81, UFBoot $=$ 50, TBE $=$ 0.82, and posterior probability from BEAST dating analyses $=$ 0.70). To facilitate discussion, we refer henceforth to this clade as “Clade I” and the remaining early-divergent lineage (labeled lineage 8 in Fig. 1) as “Clade II”. Clade II contains more than half of all extant *Bicyclus* taxa, including species from eight species groups: “*saussurei,*” “*safitza,*” “*funebris,*” “*martius,*” “*ena,*” “*dorothea,*” “*rhacotis,*” and “*angulosa.*” Each of the seven lineages of Clade I correlates with a recognised species group, except lineage 7, which contains two species groups: “*ignobilis*” and “*hewitsonii*”.

**Table 1.** Results of different BEAST dating analyses

Analysis	Relaxed clocks	Tree model	SS MLE	PS MLE	Stem age	Crown age	Clade I	Clade II
S$_{1}$	1	Yule	$-75363.23$	$-75029.17$	25.07 (21.17–28.89)	18.67 (16.14–21.53)	18.04 (15.57–20.83)	16.14 (13.85–18.77)
S$_{2}$	1	Birth–death	$-75492.79$	$-75492.54$	24.96 (21.37–28.03)	18.18 (15.63–21.12)	17.34 (14.98–19.11)	15.64 (13.49–18.04)
T$_{1}$	2	Yule	$-75117.29$	$-75119.04$	24.35 (19.94–28.85)	18.93 (15.62–21.99)	18.13 (15.08–21.21)	16.36 (13.75–19.00)
**T** $_{\mathbf{2}}$	**2**	**Birth–death**	$-74019.47$	$-73743.38$	**24.87 (20.52**–**29.13)**	**20.15 (16.23**–**23.32)**	**19.22 (15.20**–**22.30)**	**17.52 (14.04**–**20.44)**

*Notes*: The preferred results used in subsequent analyses are in bold. Parameter estimates and model performance in terms of log marginal likelihood using stepping stone sampling maximum likelihood estimation (SS MLE) and path sampling maximum likelihood estimation (PS MLE). The values under stem age, crown age, Clade I, and Clade II are median ages in Ma estimated in BEAST along with 95% credibility interval.

### Biogeography

**Figure 2.**
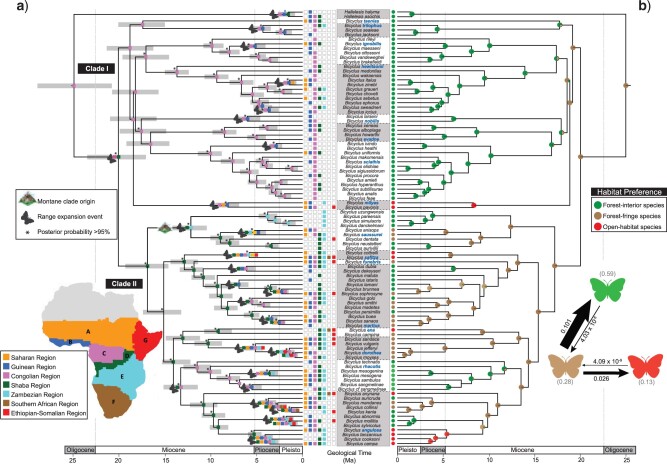
a) Bayesian divergence time estimates and historical biogeography of *Bicyclus* butterflies. Median age time tree from the BEAST dating analysis using two relaxed clocks and a birth–death speciation tree model; 95% credibility intervals are shown as gray bars on all nodes. Nodal support values are posterior probabilities (PP); nodes with PP >0.95 are labeled with an asterisk (*). Inferred ancestral ranges (coloured squares at internal nodes) are from the dispersal–extinction–cladogenesis (DEC) analysis implemented in BioGeoBEARS. Only the most likely state at each node is presented. b) On the right: results of the character optimization of host–plant preferences of *Bicyclus.* Estimation of ancestral host–plant preferences was done using the best-fit model from SecSSE analyses. The probability of habitat preference at each internal node (denoted with color-filled circles) reflects the probability of that node (i.e., ancestor) producing descendant lineages with their habitat distribution probabilities given the best-fit model and its parameters. The arrows between the colored butterflies indicate transition rates between different habitats. The width of arrows is proportional to the magnitude of the estimated transitions. The gray numbers in brackets near each colored butterfly denote the proportion of time spent in each habitat estimated from the stochastic mapping analyses implemented in phytools using 100 replicates on 200 randomly selected posterior trees. Species groups are indicated with boxes around the species name; the name of each group is in bold, blue text.

The constrained BioGeoBEARS ancestral range analysis (M2) with estimated dispersal scalar $m =$ 0.052 received the highest likelihood score (LnL $=$  $-$340.51) compared to $-$370.76 and $-$390.53 for the M1 and M0 models, respectively (Supplementary material S5 available on Dryad). Consequently, we present results of the latter analysis (M2) in Figure 2. Under the M0, M1, and preferred M2 DEC models, the Congolian region in present-day central Africa was identified as the most likely geographic origin of *Bicyclus* and its sister taxon *Hallelesis*. The initial burst of diversification that produced the eight early-divergent lineages are all inferred to have occurred in this region in the Miocene. Range expansion is the main driver of *Bicyclus’* geographic distribution on the continent (Supplementary material S6 available on Dryad). We infer range expansion from the origin to the Shaba region in the mid-Miocene, which resulted in Clade II. Some ancestors of Clade II further dispersed towards mountains in the Zambezian region of Eastern Africa, resulting in an East African endemic montane clade ($=$*saussurei* species-group). The remaining ancestors appear to have diversified mainly in the Shaba region until a late Miocene/Pliocene dispersal event back west to the Congolian region and then to rest of the continent (Fig. 2). *Bicyclus* in the Southern African and Ethiopian-Somalia regions (including the Comoros and Socotra) are results of more recent Pleistocene range expansion events of some ancestors of Clade II. Within Clade I, we infer multiple independent range expansion towards Western Africa from the geographic origin starting in the Early Pliocene.

### Modes and Tempos of Diversification

The spectral density profile of the modified graph Laplacian identified two clusters, which correspond to the two major clades identified above: Clades I and II. When we iteratively applied the eigengap heuristic to the phylogeny specifying two clusters, we found that 74 $\pm$12% of applications resulted in the same conformation of tips (and that this was the most frequently returned conformation).

**Figure 3.**
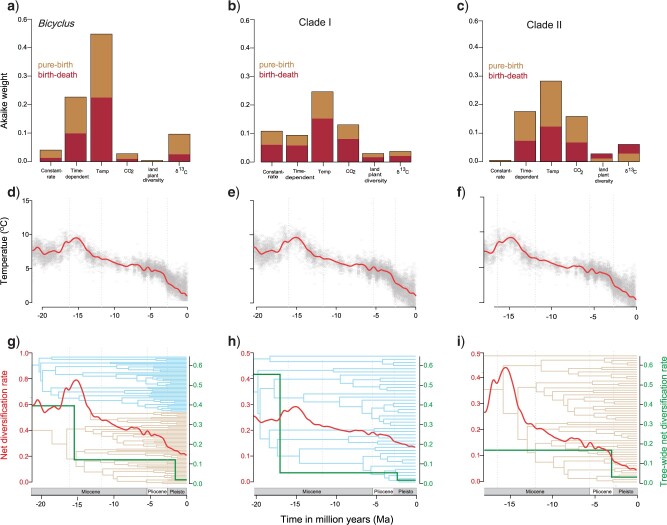
Diversification patterns of *Bicyclus* butterflies. a–c) Akaike weights for constant-rate, time-, and environment-dependent models of speciation (light brown) and speciation and extinction (dark brown). d–f) Global temperature inferred from $\delta^{18}$Oisotopes in benthic foraminifera shells recovered from marine sediments, data from Zachos et al. (2008). The y-axes are scaled to the respective crown ages of *Bicyclus*, Clade I, and Clade II, respectively, in million years ago (Ma). The red line is the median of the data points in gray. g, i) Diversification dynamics through time according to the best-fit model of positive temperature-dependent speciation (red) and best-fit episodic birth–death model (in green) for i) Bicyclus when analyzed together and for ii) Clade I and iii) Clade II, when analyzed separately.

The TreePar episodic tree-wide rate shifts analyses rejected the hypothesis of a constant diversification rate across the entire tree (Fig. 3; Supplementary Appendices S7–S9 available on Dryad). Similar results of non-constant temporal diversification were found when Clades I and II were analyzed separately. Null models of constant speciation and extinction through time were outperformed by most time- and paleo-environment-dependent models (Fig. 3; Supplementary Appendices S10–S12 available on Dryad). The best fitting episodic birth-death model for the entire phylogeny—as indicated by the LRT and AIC—is the one with two progressive decreases in diversification rates and relative extinction (turnover) at 1.5 Ma and 15.7 Ma (Fig. 3g; Supplementary Appendix S7 available on Dryad). When analyzed separately, Clade I was also best fit by a model with two decreases in diversification, one in mid-Miocene (17 Ma) and the other in Pleistocene (2 Ma). This is similar to the phylogeny of the entire genus, but with a 13-fold lower diversification rate in the mid-Miocene (compared to $\sim$4-fold genus-wide) and a further 3-fold decrease in the Pleistocene (Fig. 3h; Supplementary Appendix S8 available on Dryad). Clade II was, however, best fit by a model with a constant diversification rate of 0.089 since its origin, followed by a 3-fold downshift in rate in the late Pliocene (2.6 Ma) (Fig. 3i; Supplementary Appendix S9 available on Dryad). The TreePar analyses indicated significantly higher relative extinction (the ratio of extinction rate over speciation rate) in Clade I than in Clade II.

The relative extinction rate in Clade I was 0.503 after the origin of the clade, 0.758 after 17.0 Ma and 0.163 after 2 Ma), while in Clade II it was 0.089 after the origin with 0.143 turnover after 2.6 Ma. When compared to the entire *Bicyclus* phylogeny, the relative extinction rate of Clade I has been consistently higher at all times across the lineage’s evolutionary history. The LTT plot indicates a slowdown or near lag of diversification within the group between 15 and 8 Ma. A similar LTT plot for Clade II suggests that the events of cladogenesis leading to the extant taxa of the Clade II proceeded steadily through time since the origin of the group in the mid-Miocene about 17.5 Ma (Supplementary Appendix S13 available on Dryad).

Among the paleoenvironment-dependent likelihood models where diversification rates are functions of a time-dependent environmental variable, we found that the best fit diversification model ($\Delta $AIC >2.12; Akaike weight $=$ 41.2%) to the MCC phylogeny was a pure-birth model with a positive exponential dependence of speciation on temperature ($\lambda $(t) $=$ 0.062*exp(0.169*temperature)) with a general decrease in diversification rates towards the present (Fig. 3). Across the posterior distribution of 100 randomly selected phylogenies and including all pure-birth and birth–death-models, the same model was retained as the best fit model with 44.4% support (Supplementary Appendix S10 available on Dryad). In general, temperature-dependent models had significantly higher support compared to other paleoenvironmental variables (Fig. 3a).

We also found that the Clade I and II consensus phylogenies, albeit with a $\Delta $AIC < 2 in both cases, were best fit by a positive dependence of speciation on temperature (25.1% and 30.5% supports, respectively), similar to the entire phylogeny. Clade I is best fit with ($\lambda $(t) $=$ 0.099*exp(0.115*temperature) and Clade II with ($\lambda $(t) $=$ 0.053*exp(0.22*temperature). This was also reflected in the posterior distribution of Clades I and II phylogenies, which similarly found high support for temperature-dependence models (40.0% for Clade I and 40.3% for Clade II) (Fig. 3, Supplementary Appendices S11 and S12 available on Dryad).

Comparisons of the habitat transition rate models revealed that the most complex model (ARD) provides a significantly better fit to our character matrix than a simple one-rate model (ER, [LRT; $\chi^{2}$ (5) $=$ 22.94, $P =$ 0.0004]) and the three-rate symmetrical model, (SYM, [LRT; $\chi^{2}$ (3) $=$ 12, $P =$ 0.0073]). Therefore, the ARD transition model, where all rates differ, was used for further analyses. Bayesian stochastic mapping suggests that species inhabiting forest-interiors have arisen from ancestors living on forest-fringes and this ecological transition has occurred independently multiple times (Supplementary Appendix S14 available on Dryad). Similar transition matrices of the probability of habitat preference at each internal node were obtained using parameters that maximize the likelihood of the best supported model of the SecSSE analyses (Fig. 2).

Because the Examined-Trait-Dependent (ETD) models are strongly supported (Supplementary Appendix S15 available on Dryad), our results suggest that habitats had an important influence on  diversification. The speciation rate increased or decreased every time there was a shift in habitat preference. The speciation rate associated with forest habitats is 0.136, savannahs or open habitats have a speciation rate of 0.080, and intermediate habitats have a speciation rate of 0.211. Extinction rate was estimated at 1.16e$-$07. Interestingly, we found that speciation (branching) events are not associated with transitions between habitats. Dual inheritance is strongly supported in all possible comparable scenarios. In other words, a speciation event was not likely to happen simultaneously with a habitat shift.

The *Constrained with four rates* model best explains the evolution of habitat preferences (Supplementary Appendix S15 available on Dryad). This model outperforms the *Unconstrained* version, which suggests that *Bicyclus* species are unlikely to switch from forest directly to savanna (Fig. 2). They first adapt to an intermediate habitat, and this happens at a rate of 4.03e$-$08 evolving from forest-inhabiting ancestor, and at a rate of 4.09e$-$09 when evolving from a savanna-inhabiting ancestor. Once species are adapted to an intermediate habitat, the rate of becoming a forest species is 0.101 and the rate of becoming a savanna species is 0.026 (Fig. 2). Our results have overall support of 36.32% ($w$AIC) on the consensus tree. When repeating the analysis on 100 posterior trees, the conclusion remains the same with high statistical support ($w$AIC values range from 41.1% to 74.0%). The results of our model validation analyses suggest that habitat-dependent speciation will be detected in almost all the instances where present in our data set. We note that our method erroneously selected the ETD model when the generating model was a CTD model in 5 of 30 cases, but only selected a CTD model once in 30 cases (Supplementary Appendix S16 available on Dryad). Hence, our method will detect ETD almost always when it is present, but will occasionally detect it when it is not.

Among the key innovation models, most support is lent to the KI1 model where the subclade (Clade II) differs from the main clade (Clade I) in the carrying capacity K (Supplementary Appendix S17a available on Dryad). This was, however, not substantially different from the second and third best-fit models, KI2 and KI3 where the Clade II not only differs from the ancestral clade in the carrying capacity but also in the extinction rate or the intrinsic speciation rate. This suggests that not only did the decoupled Clade II experience a range of new niches, it was also able to colonize these niches more rapidly than would be expected from a reduction in the influence of diversity-dependence alone. The assumed key innovation is estimated to have occurred about 19.2 Mya, regardless of the scenario of the KI Model (but note that this decoupling time is restricted to lie between the stem and crown age of Clade II) (Supplementary Appendix S17a available on Dryad).

The indirect comparison of the key innovation models (KI) with the clade-wide shifts in diversification rates (SR) via the CR versions of the KI and SR models (that differ only slightly in loglikelihood, Supplementary Appendix S17b available on Dryad), suggests, however that a clade-wide shift in the clade-level carrying capacity around 9.59 Ma is a more likely explanation of the phylogenetic patterns (because of a difference in loglikelihood of almost 6 units). However, when the standard constant-rate diversification models were applied separately to Clade I and Clade II, the best-fit model was a diversity-dependent diversification process in which speciation rate decreased as diversity increased. This model received 55% and 69% support for Clade I and II respectively (Supplementary Appendix S18a,b available on Dryad).

When comparing the best-fit models for the radiation across our different macroevolutionary analyses (Table 2), an episodic birth–death model with two downshifts in diversification rates was the best-fitting model (95% $w$AIC support) describing diversification in the Clade I. The radiation within the Clade II was best explained by diversity-dependent models (65% $w$AIC support), in which speciation declines progressively as species diversity increases (Table 2).

**Table 2.** Model comparison among different diversification models for *Bicyclus* Clade I and II

Model type	**Best model**	**df**	**LogLik**	**AIC**	$\Delta $ **AIC**	***w*** $_{\mathbf{AIC}}$	$\lambda $ **1**	$\alpha $	**KI**	**Rate 1**	**Turn 1**	**ST** **1**	**Rate 2**	**Turn 2**	**ST 2**	**Rate 3**	**Turn 3**
Clade I																	
Null model	BCST	1	$-123.35$	248.70	9.44	0.008	0.130	**—**	**—**	**—**	**—**	**—**	**—**	**—**	**—**	**—**	**—**
Time-dependent	BTimeVar	2	$-122.50$	249.00	9.74	0.007	0.103	0.037	**—**	**—**	**—**	**—**	**—**	**—**	**—**	**—**	**—**
Environment-dependent	BTempVar	2	$-121.54$	247.08	7.82	0.018	0.099	0.115	**—**	**—**	**—**	**—**	**—**	**—**	**—**	**—**	**—**
**Episodic birth–death**	**BD-2 shifts**	**8**	$-111.63$	**239.26**	**0**	**0.947**	**—**	**—**	**—**	**0.013**	**0.163**	**2.0**	**0.043**	**0.758**	**17.0**	**0.548**	**0.503**
Diversity dependent	DDL	2	$-121.59$	247.18	7.92	0.018	0.22	**—**	59.7	**—**	**—**	**—**	**—**	**—**	**—**	**—**	**—**
Clade II																	
Null model	BCST	1	$-156.76$	315.52	9.50	0.006	0.146	**—**	**—**	**—**	**—**	**—**	**—**	**—**	**—**	**—**	**—**
Time-dependent	BTimeVar	2	$-152.72$	309.44	3.42	0.117	0.088	0.092	**—**	**—**	**—**	**—**	**—**	**—**	**—**	**—**	**—**
Environment-dependent	BTempVar	2	$-152.22$	308.44	2.42	0.193	0.053	0.222	**—**	**—**	**—**	**—**	**—**	**—**	**—**	**—**	**—**
Episodic birth–death	BD-1 shift	5	$-150.91$	311.82	5.80	0.035	**—**	**—**	**—**	0.027	0.142	2.6	0.171	0.089	**—**	**—**	**—**
**Diversity dependent**	**DDL**	**2**	$-151.01$	**306.02**	**0**	**0.648**	**0.30**	**—**	**70.2**	**—**	**—**	**—**	**—**	**—**	**—**	**—**	**—**

The best of each model type (null, time-dependent, environment-dependent, episodic birth–death, and diversity-dependent) are compared using their AIC scores. The best fit model (in bold) for each clade is determined by $\Delta $AIC, the difference in AIC between the model with the lowest AIC and the others; $w_{AIC}$, the Akaike weight of each model, expressed as a percentage. df $=$ degree of freedom; AIC $=$ Akaike Information Criterion; $\lambda $  $=$ speciation rate; $\mu =$ extinction rate; BD $=$ birth–death. The best-fit models are BCST $=$ Yule (pure birth), BTimeVar $=\lambda $ varies with time, BTemp.Var $=\lambda $ varies with Temp, BD-2 shifts $=$ BD with two shifts in diversification rates, DDL $=\lambda $ declines linearly with diversity with no $\mu $. Other parameter estimates are denoted as follows: rate, net diversification rate (speciation minus extinction); Turn $=$ Turnover (extinction over speciation); ST $=$ Shift time, in which “Rate 1” denotes the diversification rate and “Turnover 1” is the turnover, both inferred between Present and the shift time 1.

## Discussion

### Origin and Modes of Speciation

Our analyses provide novel insights into the evolution of Africa’s terrestrial biodiversity in the Cenozoic. *Bicyclus*, Africa’s most species-rich satyrine butterfly genus, originated in the Congolian rainforest block in Central Africa around the late Oligocene (*ca.* 25 Ma) when it diverged from its sister taxon *Hallelesis*. Initial diversification of extant *Bicyclus,* however, began *ca.* 5 million years later when eight major extant lineages diverged in rapid succession. The antiquity and rapidity of these divergences in the Miocene hampered attempts to untangle the precise topology of this portion of the tree with confidence. Ancestral range and habitat reconstruction analyses suggest that these early rapid divergences all occurred within ancient Central Africa forests (Fig. 2).

Spectral density profile analyses reveal two diversification patterns within *Bicyclus*: 1) the seven early-divergent lineages comprising Clade I diversified recently in the late Miocene to early Pliocene (*ca*. 8–2.5 Ma); and 2) Clade II diversified steadily when the lineage split from the rest of *Bicyclus* after the mid-Miocene (*ca*. 17.5 ma), reaching its current size of >50 extant species. The hypothesis that Clade I and Clade II diversified under two distinctly different modes is further supported by ancestral habitat state and geographic range reconstructions. Except for the *milyas-*group comprising *B. milyas* and *B. pavonis*, all extant taxa in Clade I are strict forest-interior adapted species and most live in Western and West-Central Africa forests (Fig. 2). Our ancestral range and habitat analyses reveal that Clade I has undergone extensive *in situ* diversification in Congolian forests in West-Central Africa, and only later in the late Miocene—early Pliocene did their ranges expand to include forests further west and east of its provenance (Fig. 2). Clade II is inferred to have diversified largely in Eastern Africa, outside the distribution of Clade I, and later dispersed back to the inferred geographic origin of the genus and to other regions of Africa. Unlike the forest-loving species of Clade I, extant species of Clade II live in a range of habitat types and include forest specialists, savannah specialists, and habitat generalists (intermediate or forest-savannah mosaic adapted species). They are widely distributed across Africa (Fig. 2), suggesting a more resilient common ancestor.

### Forest Refugia in Central Africa Acted as “Museums” of Cenozoic Biodiversity

We hypothesize that the early, rapid divergences within the genus resulted from fragmentation of ancestral populations into forest refugia in the early- to mid-Miocene. Paleoenvironmental data suggest that vegetation of the early Miocene was dominated by tropical and subtropical forests (Zachos et al. 2008). In Africa, even the land covered by the present-day Sahara Desert was forested (Barry et al. 1991; Coetzee 1993; Jacobs 2004; Plana 2004; Micheels et al. 2009) and the ancestors of modern *Bicyclus* were likely widely distributed across Africa in these putative ancient forests.

However, drastic global cooling in the mid-Miocene, triggered by the closure of the Tethys Sea and associated changes in tropical oceanic currents (Zachos et al. 2001; Rommerskirchen et al. 2011; Zhang et al. 2011), favored grassland expansion and contraction of dense canopy forests (Jacobs 2004; Micheels et al. 2009; Senut et al. 2009). This transformed the continent into a mosaic of isolated refugial forests separated by savannah (Jacobs 2004; Plana 2004). Consequently, rainforests in Africa were mostly fragmented into relatively small patches in upland and lowland watersheds (Coetzee 1993; Plana 2004). Many plants and animals presumably went extinct if they could neither tolerate nor adapt to the new, drastically cooler temperatures and associated biomes nor disperse to climatically favorable areas (Morley and Richards 1993). For example, both modern and paleofloras provide evidence that many plant groups in tropical Africa became taxonomically depauperate compared to those in more climatically buffered regions such as Southeast Asia and Madagascar (e.g., Pan et al. 2006).

We found support for a significant, tree-wide decline in diversification rates during the mid-Miocene—particularly for lineages in Clade I—which strongly correlates with abrupt changes in global temperatures (Supplementary Appendices S7–S9 available on Dryad, Fig. 3g,h) and is furthermore best described by a diversification model of positive temperature-dependence (Fig. 3a–c). Results of our episodic diversification analyses also indicate higher relative extinction rates in the entire phylogeny during this period compared to the late Miocene and Pliocene (turnover 0.33 vs. 0.14) (Supplementary Appendix S7 available on Dryad). This suggests that many ancestral populations went extinct during this harsh climatic epoch and that only those “trapped” in more stable forest refugia survived, such as the ancestors of the eight early-divergent lineages recovered in our phylogeny. Most of the postulated Miocene forest refugia in Africa that might have provided relatively stable forest environments (“museums”) for the surviving ancestors inferred to have been in Central Africa (Plana 2004). Coincidentally, ancestral range estimation analyses identified Central Africa as the origin of the genus and of the ancestors that gave rise to all eight early-divergent lineages, including the MRCA of Clades I and II (see Fig. 2). These refugial forests therefore acted as “museums” harboring ancient lineages of that later expanded their ranges and diversified into the modern taxa of the group.

The phylogenetic relationships among the eight early-divergent lineages are poorly resolved, even when inferred with ten phylogenetically informative protein-coding markers frequently used in butterfly systematics (Wahlberg and Wheat 2008). These loci recover deep divergences across a range of butterfly genera and tribes (e.g., Aduse-Poku et al. 2015; Chazot et al. 2019; Wahlberg et al. 2009). However, rapid population fragmentation, which is strongly supported by our best-fitting episodic birth–death model, allows little time for accumulation of synapomorphic changes in the genome (fixation) between successive divergences (Kodandaramaiah et al. 2010). These short intervening periods would further obscure phylogenetic signal among the resultant daughter taxa, making it difficult to resolve their evolutionary relationships (Wiens et al. 2008). This is likely why we were unable to untangle the successive events of diversification among the early-divergent lineages with confidence. It is unlikely that low taxon sampling could account for the unresolved basal splits as *ca.* 92% of all known species were included in the analyses.

The episodic birth-death model, which received 95% support against all competing best-fit macroevolutionary models (Table 2), reveals a significantly high (0.76) background extinction within Clade I beginning at the peak of the MMCO,* ca.* 18–15 Ma (Bohme 2003; Sun and Zhang 2008). This period marked a precipitous 13-fold decline in diversification rates within the clade *ca.* 17 Ma (Fig. 3; Supplementary Appendix S8 available on Dryad) from 0.55 to 0.04. The long stem branches subtending the relatively recent 3-9 Ma crown diversification of the seven early-divergent lineages of Clade I are consistent with mid-Miocene periods of high extinction (Supplementary Appendix S13 available on Dryad). Interestingly, with the exception of the ditypic *milyas* group (lineage 8 in Fig. 1), all inferred diversification events within Clade I occurred in forest ecosystems (Fig. 2b).

The late Miocene to early Pliocene (9–3 Mya) is thought to have been characterized by intermittent moist climates in Africa, resulting in alternating expansion and contraction of forested habitats (Morley 2000; Plana 2004). We hypothesize that the early-divergent lineages of Clade I responded to this putative paleoclimatic oscillation and diversified by extending their ranges beyond their original postulated refugia locations, when environmental conditions were favorable (i.e., wetter and warmer). Intervening cool/dry phases are believed to have further isolated the populations of this forest-dwelling clade as ancients forests contract in response to the prevailing climate change. Subsequent adaptations of these populations to isolated forest fragments and local host plants likely set the stage for ecological speciation via divergent natural selection, or alternatively via neutral processes such as drift if niches remained conserved for sufficient time (Schluter 2009; Nosil 2012). The Neogene climatic oscillations are known to have spurred major diversification in other taxa such as other butterfly groups (Aduse-Poku et al. 2009; Van Velzen et al. 2013), galagoes (Pozzi 2016), birds (Nguembock et al. 2009), frogs (Wieczorek et al. 2000), and plants (Plana 2004; Couvreur et al. 2011).

### Diversification in a Changing Mio-Pliocene Climate and Biome

The second mode of diversification describes the rapid radiation of a single parent lineage (MRCA of Clade II, lineage 8 in Fig. 1), which has over 50 extant taxa living in a variety of habitats, including several geographically widespread species. It seems plausible that some ancestral populations of Clade II differentiated into species through directional selection in response to the prevailing climatic changes that began around 17 Ma when the clade started diversifying. Temperature- and atmospheric CO$_{2}$-dependent models received 40% and 23% support, respectively, as plausible explanations of diversification within this clade (Fig. 3; Supplementary Appendix S12 available on Dryad). However, a comparison of model performance across all macroevolutionary analyses (Table 2) significantly supports diversity-dependent processes as the main driver of diversification within the Clade II (65% $w$AIC, $\Delta $AIC>2). This suggests that species-intrinsic (biotic) interactions might have played a substantial role in the clade’s diversification.

Clade II is inferred to have split into two strongly supported lineages that differ ecologically and likely evolved along different trajectories. One route led to the fully montane *saussurei* group (Aduse-Poku et al. 2017), which are primarily distributed in the mountains of Eastern and Eastern Central Africa (Fig. 2). The other species-rich clade comprises mainly lowland species with a diverse range of habitat preferences, including *B. anynana* and other generalist species. We estimate the divergence time of these two lineages within the Clade II to be around 17.5 Ma (95% HPD 14.4–21.0), which coincides with the onset of uplift in the western branch of the East African Rift System (Roberts et al. 2012; Wichura et al. 2015). This putative geological event is believed to have isolated the MRCAs of the Clade II into two distinct subclades: one fully submontane and the other confined largely to lowlands. The orogeny of the East African Plateau (EAP) continued throughout the Miocene (Roberts et al. 2012; Wichura et al. 2015) with ensuing significant climatic and environment changes (Cerling et al. 1997), including an early expansion of C$_{4}$ plants in Eastern Africa *ca.* 10 Ma (Uno et al. 2016), and expansion of grass-dominated savannah biome that started in mid-Miocene (*ca.* 16 Ma) and became widespread in the late Miocene (*ca.* 8 Ma) (Jacobs 2004; Edwards et al. 2010). The final uplift of the EAP is estimated to be in the Plio-Pleistocene (Sepulchre et al. 2006; Roberts et al. 2012; Wichura et al. 2015). We hypothesize that these geological events and the regional aridification and related environmental changes they induced further drove habitat fragmentation and ultimately spurred diversification within the montane lineage (Cerling et al. 1997). Similar patterns of montane speciation have been found in plants (Dagallier et al. 2020), birds (Roy et al. 2001) and, outside Africa, there are similar reported cases from the New Guinea (Toussaint et al. 2014), the Himalayan–Tibetan mountains (Condamine 2018; Condamine 2018b), and the Andes (Hoorn et al. 2010).

Radiation within the remaining taxa of the Clade II, although rapid, allowed confident inference of tree topology. The onset of diversification within this clade is estimated at 15 Ma, around the end of the MMCO. During the MMCO, eastern Africa became drier, causing forest habitats to become grassland (Coetzee 1993; Jacobs 2004) ostensibly in response to the rapid decline in global temperatures after the MMCO. Unlike Clade I, in which clade-wide diversification rates declined considerably during this period, Clade II diversified at a constant rate of 0.17 from its origin and during this period until a decrease in the diversification rate during the Pliocene (2.6 Ma) (Fig. 3i, Supplementary Appendices S9 and S13c available on Dryad). This suggests that the Clade II was either less impacted by the abrupt climatic changes of this period or better able to deal with them, consistent with the Red Queen Hypothesis (Van Valen 1973). The latter explanation appears more plausible because extant species in this group are adapted to a broad range of habitats including various forest types, riverine forests and thickets, through woodlands, bushlands, grasslands, to rocky, semi-arid and sparsely wooded savannah areas (Condamin 1973; Larsen 2005). Our (trait)state-dependent analyses demonstrate the strong influence of habitat diversity on diversification (Supplementary Appendix S15 available on Dryad). Lineages that inhabited forests and transitioned into living in forest-fringe habitats speciated more rapidly (0.13–0.20 per lineage per million years). These results suggest ecological release: a trait related to successful colonization of a novel habitat led to a burst of diversification in the absence of competition (Yoder et al. 2010; Halali et al. 2020). The diversity-dependent model for this clade received an overwhelming (95%) support further supporting the role of ecological release (Table 2).

Many species in Clade II have morphologically distinctive seasonal forms, particularly species inhabiting habitats with climatically different dry and wet seasons (Brakefield and Larsen 1984; Windig et al. 1994). Most species in Clade I lack this seasonal polyphenism, suggesting that this phenotypic variability in Clade II evolved in response to the seasonally fluctuating environment during the Neogene (Bohme 2003). The adaptive value of different seasonal phenotypes has been well studied in the model species *B. anynana* (Brakefield and Frankino 2009; van den Heuvel et al. 2013; Prudic et al. 2015). However, it is not possible to determine whether seasonal polyphenism is ancestral or derived in Clade II. Similar seasonal polyphenism seems to have evolved convergently in the distantly related Asian satyrine genus *Mycalesis* (Brakefield and Larsen 1984) and in *Melanitis leda* (Brakefield and Larsen 1984), *Junonia coenia* (Rountree and Nijhout 1995), and *Araschnia levana* (Windig and Lammar 1999), suggesting either convergent evolution in response to environmental seasonality (Shapiro 1980) or occasional expression of a conserved trait in butterflies (van Bergen et al. 2017; Bhardwaj et al. 2020).

A recent study (Louca and Pennell 2020) suggested that an infinite number of alternative diversification scenarios can be generated from any given extant time-tree. More recent scrutiny of this assertion (Morlon et al. 2020; Helmstetter et al. 2021) concludes that diversification rates (speciation minus extinction) can be modeled, even if estimating extinction rates in isolation remains challenging. The hypothesis-driven nature of this study suggests that the diversification scenarios presented are the most likely, as they are temporally compatible with multiple data types that cross-validate each other.

### Host Plant Use and Bicyclus Evolution

The evolutionary success of Clade II seems tightly linked to their ability to adapt to the Miocene climate transition from warm and humid environments to dry and seasonal habitats. Consequently, it is the only early-divergent lineage that includes widespread, eurytopic species adapted to all habitat types (Fig. 2). It is puzzling why most of the seven other early-divergent lineages in Clade I produced only a few extant species. The SecSSE analyses strongly favors Dual inheritance of habitat preference in which both daughter species inherit the same habitat preference as the parent species (Supplementary Appendix S15 available on Dryad; Fig. 2b). Thus, niche conservatism in Clade I might have constrained diversification because their host plant ranges and preferred habitats contracted during the Miocene, and this lineage was unable to adapt.

The widely held viewpoint that *Bicyclus,* like most satyrine nymphalids, feed on grasses (Poaceae) (Peña et al. 2006) may not be accurate for the majority of the species, especially for forest-adapted taxa in Clade I. Attempts to culture many Clade I species in the laboratory have always been unsuccessful, presumably because they feed on a narrow selection of unknown host plants (KAP, OB, pers. obs.). Sourakov and Emmel (1997) tried to induce a dozen *Bicyclus* and *Hallelesis* species to lay eggs on several perennial domesticated grasses in captivity. None of the Clade I species included in the study accepted the grasses offered (*B. larseni, B. procora, B. xeneas,* and *B. taenias*), suggesting they are not generalist herbivores of Poaceae as larvae. They may not feed on grasses at all, which might explain the dearth of host–plant records for this group of butterflies despite their abundance and popularity among collectors: field biologists may be looking on the wrong plants to find eggs and larvae. The vast majority of Satyrinae feed only on monocots, and the only host plant records for species in Clade I, include Zingiberaceae and Marantaceae for the *hewitsonii*-species group. *Bicyclus iccius,* also a member of the *hewitsonii*-species group has been recorded on Poaceae (Ackery et al. 1995), but Larsen (2005) considers this record to be dubious.

As expected of taxa with specialized host–plant associations, allopatric differentiation is presumed to be the dominant mechanism of divergence since colonization of novel environments requires the presence of suitable hostplants. Recent taxonomic scrutiny tends to find that isolated populations in Clade I are novel species (Brattström et al. 2015; Brattström et al. 2016). This suggests that ecological speciation via hostplant specialization may play a role in the diversification of this group—a hypothesis that can be tested when more hostplant data are available. Extant species of Clade II, however, have only been observed feeding on Poaceae (Condamin 1973; Larsen 2005). Most available host plant records for *Bicyclus* are documented from species in this clade, giving the general impression that *Bicyclus* are grass feeders. However, this clade is just one of eight early-divergent *Bicyclus* lineages emerging in the early Miocene.

## Conclusion

We present a robust, time-calibrated phylogenetic hypothesis for the African butterfly genus *Bicyclus* documenting a series of rapid early divergences beginning 5 Ma after divergence from its sister genus, *Hallelesis*. The first bifurcation within *Bicyclus* resulted in two clades with contrasting habitat requirements that responded differently to environmental change. Most of the 100$+$ extant species result from the success of just one of its eight early-divergent lineages. This group, which we call Clade II, was able to climate and habitat changes in the Miocene, when grasslands expanded and forest contracted, while the other seven early-divergent lineages, comprising Clade I, remained forest specialists. The tempo and mode of this diversification has many key attributes of adaptive radiation (Schluter 2009). The most important of these is rapid speciation of the group in response to suspected ecological divergence among the lineages of common ancestors. The other prerequisite of adaptive radiation, namely a correlation between phenotype and the environment, provides a basis to investigate fitness advantages of trait values to their corresponding environments. A single species, *B. anynana*, has been a model for studying development and evolution for almost four decades. The phylogeny and macroevolutionary hypotheses provided here now serve as a scaffold to extend the rich arsenal of ecological and evolutionary information gained from this species to the remainder of the taxa in the radiation. Allopatric divergence has also clearly been important, especially along the backbone of the tree, most probably in response to mesic forest fragmentation during the peak of the mid-Miocene global warming. Lastly, although we do not assume to have captured all the factors and processes that culminated in radiation of *Bicyclus* on the African continent, our results provide strong arguments for the influence of biotic and abiotic factors on the diversification of *Bicyclus* and represent a step forward in understanding the mechanisms that led to the rich extant biodiversity of Africa.

## Supplementary Material

Data available from the Dryad Digital Repository: http://dx.doi.org/10.5061/dryad.qz612jmcb.
